# Design of a Broadband Band-Pass Filter with Notch-Band Using New Models of Coupled Transmission Lines

**DOI:** 10.1155/2014/238717

**Published:** 2014-08-28

**Authors:** Navid Daryasafar, Somaye Baghbani, Mohammad Naser Moghaddasi, Ramezanali Sadeghzade

**Affiliations:** ^1^Department of Electrical Engineering, Dashtestan Branch, Islamic Azad University, Dashtestan, Iran; ^2^Department of Electrical Engineering, Bushehr Branch, Islamic Azad University, Bushehr, Iran; ^3^Faculty of Engineering, Science and Research Branch, Islamic Azad University, Tehran, Iran; ^4^Faculty of Electrical and Computer Engineering, K.N.Toosi University of Technology, Tehran, Iran

## Abstract

We intend to design a broadband band-pass filter with notch-band, which uses coupled transmission lines in the structure, using new models of coupled transmission lines. In order to realize and present the new model, first, previous models will be simulated in the ADS program. Then, according to the change of their equations and consequently change of basic parameters of these models, optimization and dependency among these parameters and also their frequency response are attended and results of these changes in order to design a new filter are converged.

## 1. Introduction

**Figure 1:**
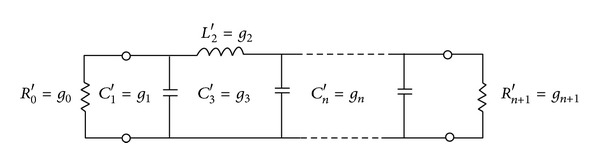
Circuit model and primary template of a low pass filter [1].

In this paper, before presenting new models of coupled transmission lines it is necessary to present and analyze the general method of designing band-pass filters. According to the general method of designing band-pass filters in [1], using the template of integrated elements, first a low pass filter is designed and then the equivalent band-pass filter which uses coupled transmission lines will be designed and analyzed.  Figure 1 shows a primary example of a low pass filter [1].

In order to acquire the parameters and integrated elements of a band-pass filter at the resonance frequency *ω*_0_ based on the parameters and integrated elements of a low pass filter according to its parallel or serial structure, two different parameters will be defined. One of the parameters which is defined at the central resonance frequency of the filter is the slope parameter. For any serial resonator the reactance slope parameter is defined by [1]

(1) $$\begin{array}{c} \alpha ={\frac{\omega_{0}}{2}\frac{dX}{d\omega }|}_{\omega_{0}}\;\text{Ohm}. \end{array}$$

In (1),* X* is the reactance of the resonator.

Similar to this parameter, for a parallel resonator the susceptance slope parameter is defined by this equation [1]

(2) $$\begin{array}{c} \gamma =\frac{\omega_{0}}{2}{\frac{dB}{d\omega }|}_{\omega_{0}}. \end{array}$$

**Figure 2:**
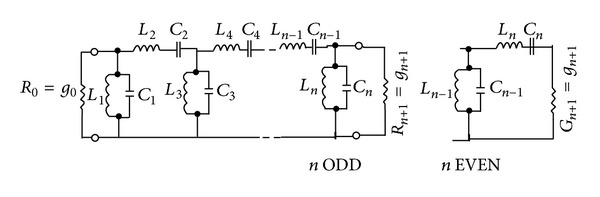
Circuit model of the band-pass filter derived from the primary template of a low pass filter.

In (2),* B* is the parallel resonator susceptance. After designing the low pass filter and determining the integrated elements used in its circuit model, the circuit model of the band-pass filter can be obtained using the primary template. As can be observed in Figure 2, in the circuit model of the band-pass filter derived from the primary template of the low pass filter, parallel and serial resonators have been used. In order to calculate the values of the integrated elements in the parallel and serial resonators, in the circuit model of the band-pass filter which has been designed based on the values of the integrated elements used in the primary template of a low pass filter, the reactance and susceptance slope parameters are used.

The equations which present the relations between the integrated elements of the band-pass and low pass filters for parallel and serial resonators are as follows:

(3) γj=ω9Cj=1ω0Lj=ω1′gjW,αK=ω0LK=1ω0CK=ω1′gKW.

In (3),* W* is the relative bandwidth and *ω*_0_ is the central resonance frequency for the band-pass filter.

In [1, 5], the procedure of deriving the band-pass filter equivalent to the low pass filter has been thoroughly explained. Figure 2 shows the band-pass filter equivalent to the low pass filter shown in Figure 1 which has been derived directly from the primary template of the low pass filter.

Acquiring the equivalent structure of the transmission lines with the structure of the band-pass filter shown in Figure 2 at microwave frequencies is difficult and complicated. To solve this problem, admittance and impedance transformers are usually used in the design of band-pass filters [1].

## 2. The Design of Band-Pass Filters Using Coupled Resonators

**Figure 3:**
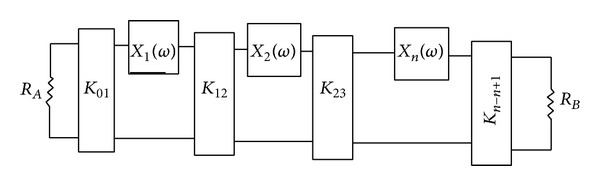
Circuit model of the band-pass filter using* K* transformers and serial reactance.

In this section we will analyze the design of a band-pass filter which has coupling between transmission lines in its structure. For the design of the band-pass filter which utilizes coupling in its structure, we can consider a circuit model similar to what is shown in Figure 3. The only difference in this state is that the serial reactance shown in circuit model of Figure 3 determines the amount of coupling between the coupled lines used in the structure of the filter.

In the design of the primary model of band-pass filters and similar to the case without coupling, in this case we can use integrated elements in the circuit model of the band-pass model to show coupling.

**Figure 4:**
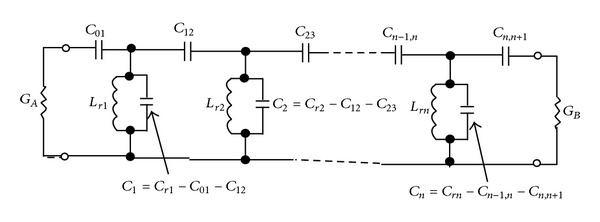
Circuit model of a band-pass filter with electric coupling between the transmission lines used in its structure.

Considering the model of Figure 3 and by replacing the* K* transformers with integrated elements and determining the amount of coupling between the transmission lines and the coupled capacitors, we can create a model similar to what is shown in Figure 4 for a band-pass filter which has coupling in its structure.

It must be noted that, in circuit structure of Figure 4, the *C*_*rj*_ capacitors are the main factor in determining the resonance frequency and susceptance slope of the resonators.

## 3. Simulations of Broadband Band-Pass Filters in Recent Studies

The two band-pass filters which will be studied in this paper are from the most recent research made in the past few years. One of them has been designed using stepped-impedance resonators (SIR), and the other has been designed using multiple-mode resonators (MMR), and the new model proposed in this paper is an improved model using these two resonators.

It must be noted that the greatest deficiency of these two filters is the lack of notch and by presenting a new model in the next section this issue will be solved.

### 3.1. Three-Section MMR

**Figure 5:**
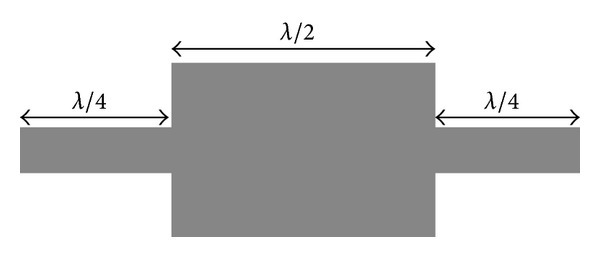
The structure of a MMR.

One of the most important resonators which has gained the attention of designers of passive microwave elements is MMR resonators, and many band-pass filters have been designed based on these resonators [6–10].  Figure 5 shows the structure of a MMR, which is the main resonator used in the design of such broadband filters.

**Figure 6:**
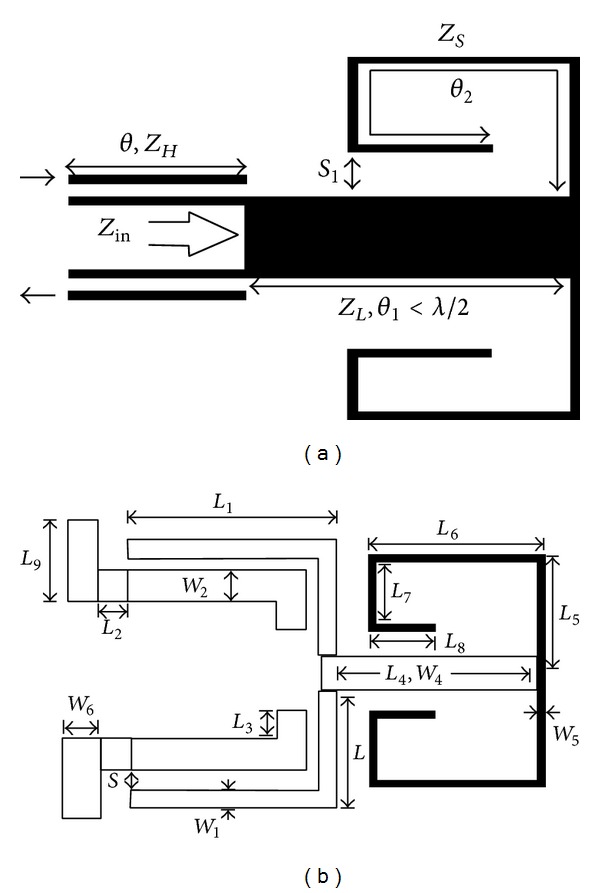
(a) Structure of the band-pass filter designed based on the MMR resonator shown in Figure 5. (b) The structure based on optimized values.

In the next step, based on the MMR resonator presented in Figure 5, a broadband band-pass filter which has been designed and proposed will be simulated.  Figure 6 shows the structure of this band-pass filter designed based on the MMR resonator.

**Figure 7:**
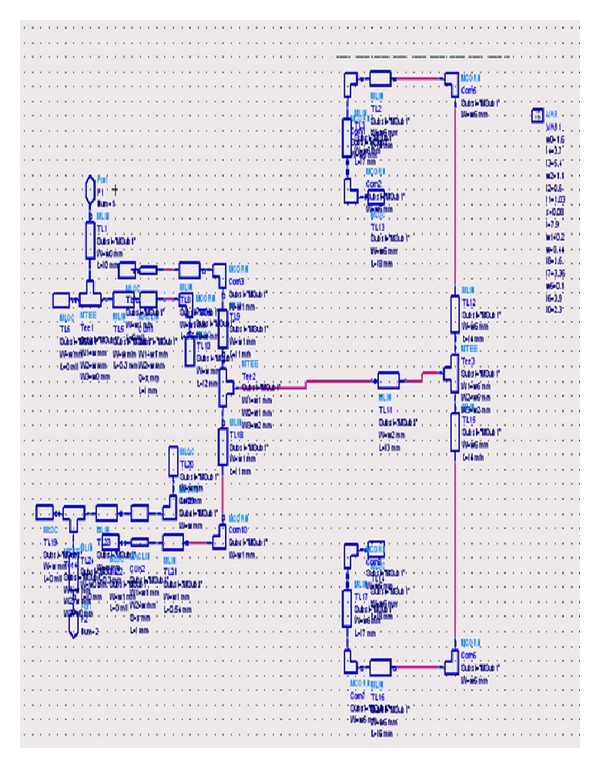
Implemented structure of the filter of Figure 6 in the* S*-parameters workspace of the ADS program.

As can be observed from the structure of the band-pass filter presented in Figure 6, the presented filter includes one MMR resonator and two coupling sections where the length of the coupling lines is equal to the two lateral sections of the filter. Similarly to the band-pass filter designed using SIR resonators in the next section, the structure of the filter presented in Figure 6 will first be implemented in the* S*-parameters workspace.  Figure 7 shows the implemented structure of the filter of Figure 6 in the* S*-parameters workspace of the ADS program.

After implementing the structure of this filter, all of its parameters will be selected in order to have an acceptable frequency response in the ADS software which are based on the values given in Table 1.

**Figure 8:**
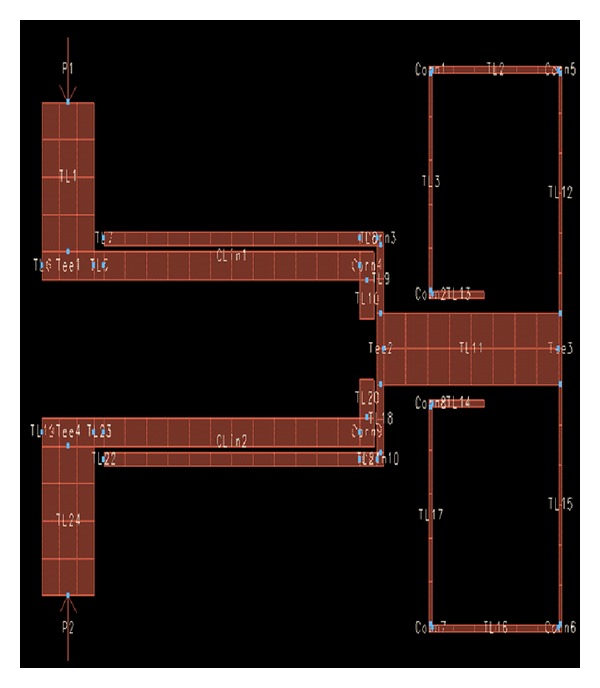
Structure of the broadband band-pass filter designed using a MMR resonator in the workspace of the ADS program.

After selecting the optimized values for the parameters of the designed filter, its structure will be taken to the workspace of the program.  Figure 8 shows the structure of this broadband band-pass filter designed using a MMR resonator in the workspace of the ADS program.

**Figure 9:**
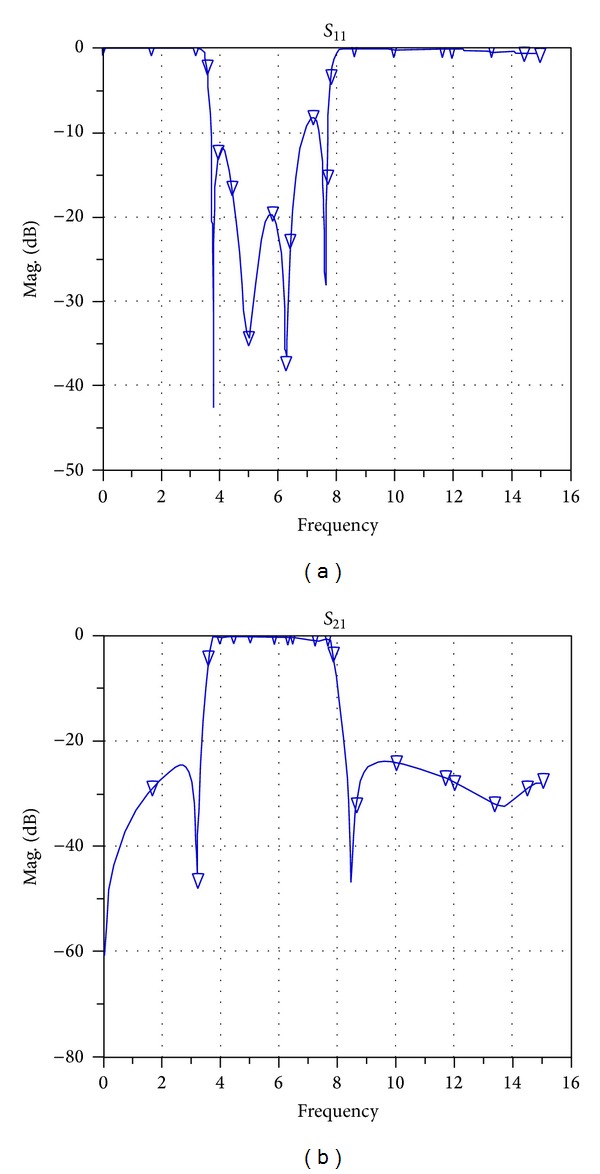
Simulated frequency response of the broadband band-pass filter designed using a MMR resonator in the workspace of ADS versus return losses (*S*_11_) and insertion losses (*S*_21_).

After implementing the filter, the structure of this filter is implemented on a substrate with a thickness of 0.508 mm and dielectric constant of 2.2 and the frequency response is simulated in the range of 0–16 GHz.  Figure 9 shows the simulated frequency response of the broadband band-pass filter designed using a MMR resonator in the workspace of ADS versus return losses (*S*_11_) and insertion losses (*S*_21_).

**Figure 10:**
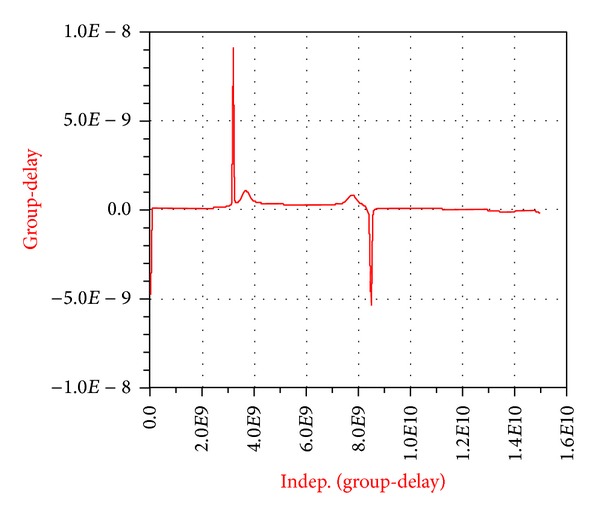
Group-delay of the broadband band-pass filter designed using a MMR resonator.

As shown in Figure 9, the values of the* S*-parameters have been presented which means that the filter phase parameter must also be analyzed. Usually the phase parameter (*S*_21_) is presented as the phase delay. In this paper and in Figure 10 this parameter is shown as group-delay.

It must be noted that one of the main positive characteristics of filters designed using MMR resonators compared to filters designed using SIR resonators is that the first harmonic is far from the central resonance frequency and also their greater bandwidth. In order to show this fact in the next step, the frequency response of the filter in a greater range and both losses will be shown in a diagram.

**Figure 11:**
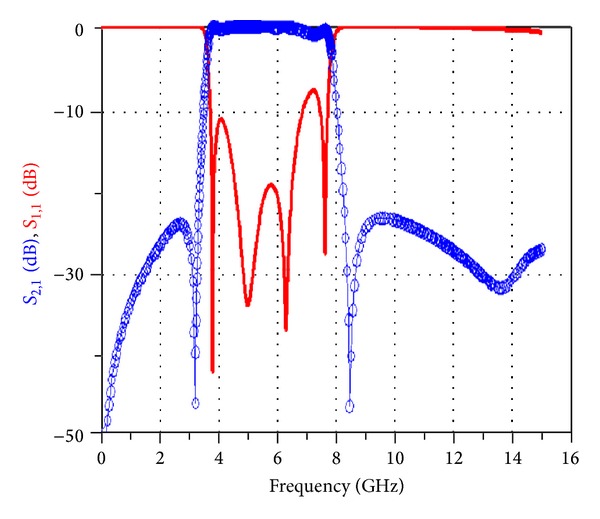
The simulated frequency response of the broadband band-pass filter designed using a MMR resonator in the workspace of ADS versus return losses (*S*_11_) and insertion losses (*S*_21_) in a range of 0–16 GHz.

Figure 11 shows the simulated frequency response of the broadband band-pass filter designed using a MMR resonator in the workspace of ADS versus return losses (*S*_11_) and insertion losses (*S*_21_) in a range of 0–16 GHz.

### 3.2. Two-Section SIR Resonator

Another important resonator which has been studied by designers of passive microwave elements is the SIR resonator [2, 11, 12].

**Figure 12:**
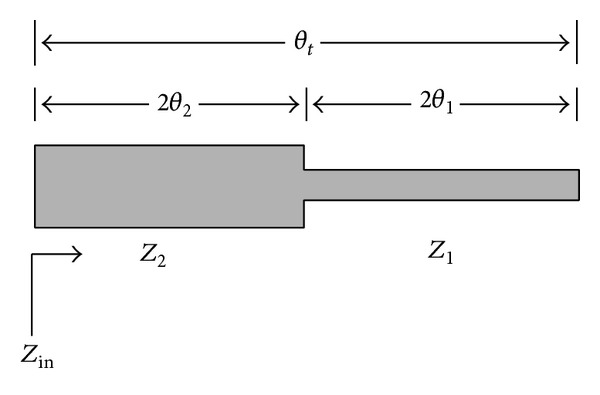
Structure of the asymmetric two-section SIR used in [2].

The broadband band-pass filter designed in [2] is chosen.  Figure 12 shows the structure of the asymmetric two-section SIR used in this reference.

**Figure 13:**
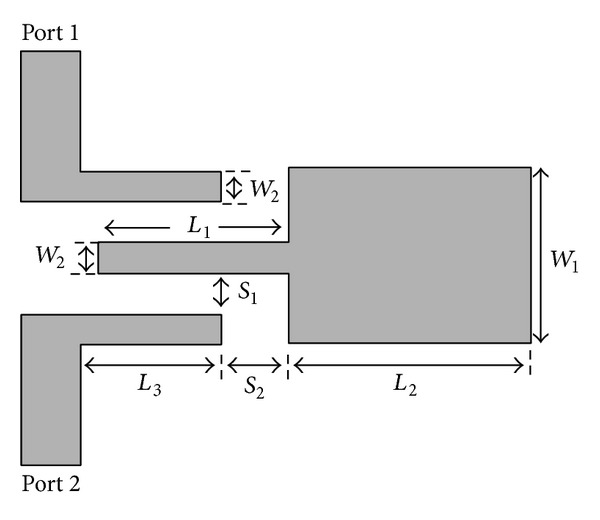
Structure and model of the broadband band-pass filter presented in [2].

Figure 13 shows the structure and model of the broadband band-pass filter presented in [2].

In [2] and based on the structure presented in Figure 13, a broadband band-pass filter has been designed, analyzed, and built. The structure of this filter has been implemented on a Duroid 5880 substrate with a thickness of 0.787 mm and a dielectric constant of 2.2.

**Figure 14:**
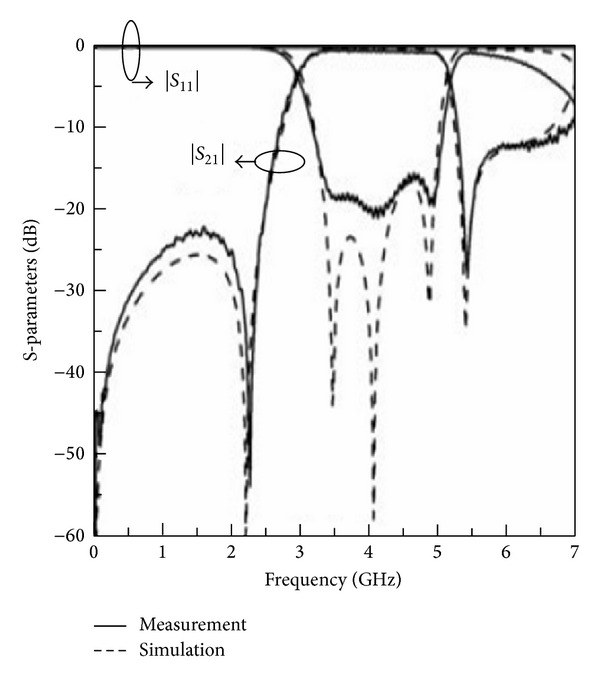
Comparison between the simulated and measured frequency response of the band-pass filter designed using the given asymmetric SIR.

Similarly, Figure 14 shows a comparison between the simulated and measured frequency response of the band-pass filter designed using the asymmetric SIR given in Figure 12.

**Figure 15:**
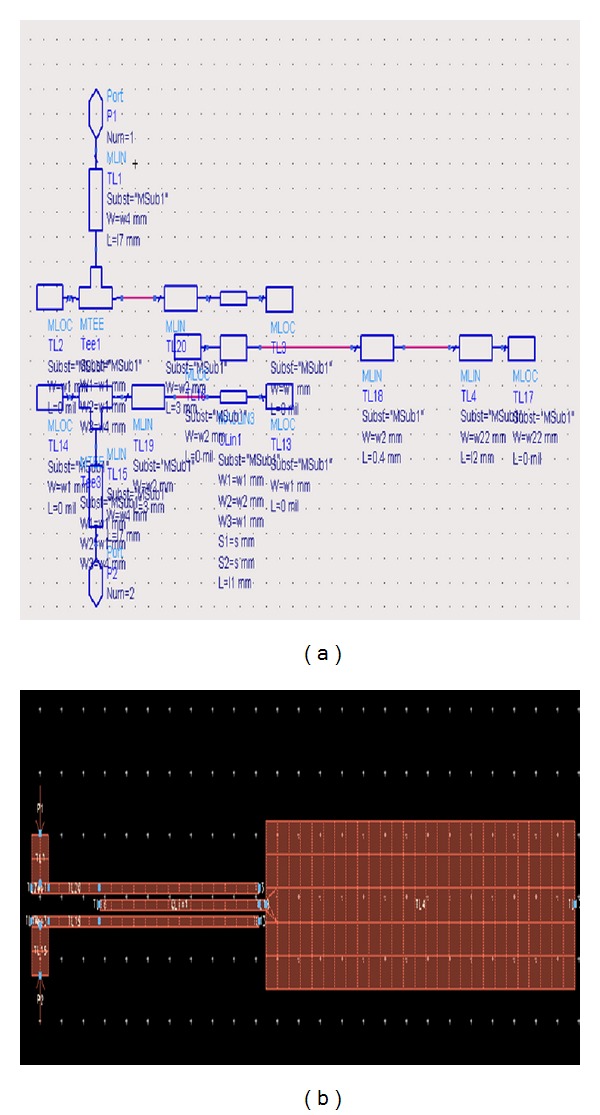
Structure of the broadband band-pass filter of [2] in the workspace of ADS.

Figure 15 shows the implemented broadband band-pass filter using values given in Table 2, in the* S*-parameters workspace of the ADS program, which has been formed using 15 microstrip transmission lines.

**Figure 16:**
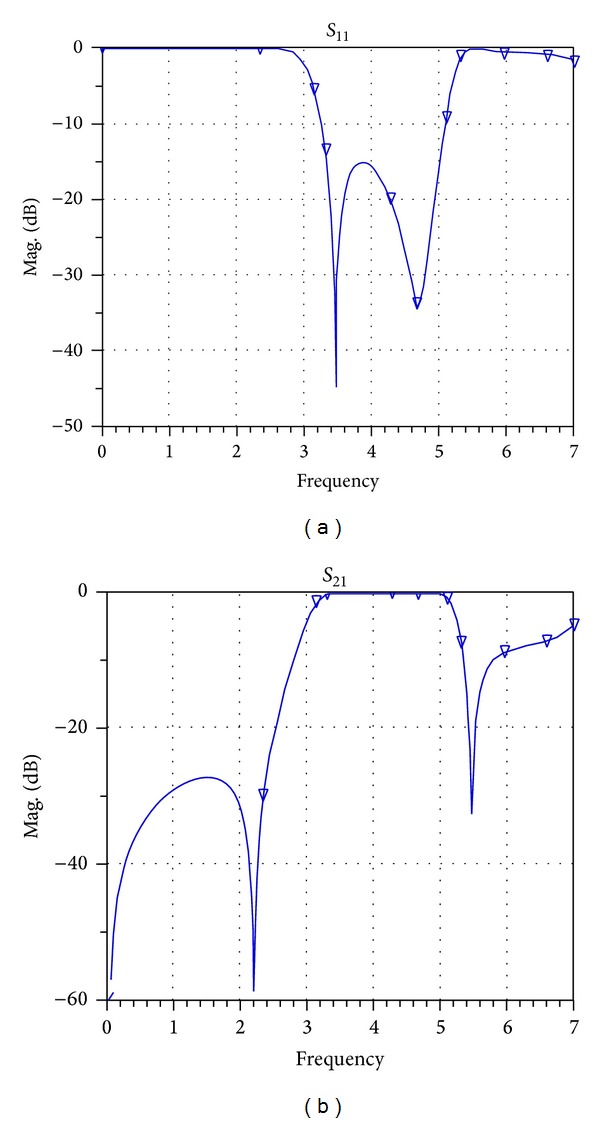
Frequency response of the simulated filter of [2] in the workspace of ADS plotted versus (a) return losses (*S*_11_) and (b) insertion losses (*S*_21_).

**Figure 17:**
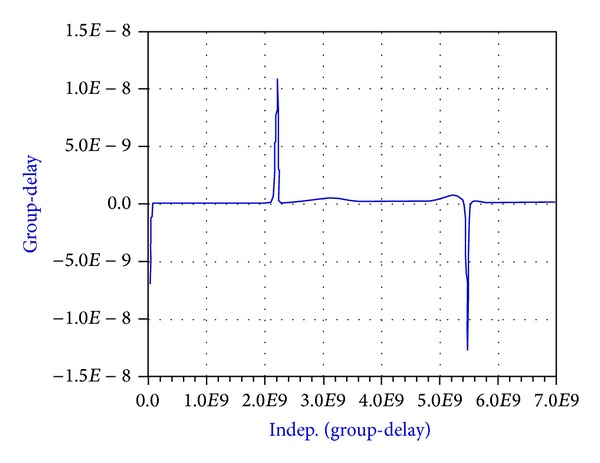
Frequency response of the simulated filter of reference in the workspace of ADS versus group-delay.

After implementing the filter in the workspace of ADS, the structure of this filter is implemented on a substrate with a thickness of 0.787 mm and a dielectric constant of 2.2 and then the frequency response in the range of 0–7 GHz is simulated.  Figure 16 shows the frequency response of the simulated filter in the workspace of ADS plotted versus return losses (*S*_11_) and insertion losses (*S*_21_). In this paper and in Figure 17, the parameter (*S*_21_) is shown as group-delay.

As was noted, one of major deficiencies of the reference band-pass filter was the first harmonic being close to the filter central frequency, and in the frequency response of this filter and in [2] the existence of this harmonic was not mentioned. In order to show this harmonic and to study its limitations, the frequency response of this filter is simulated in a wider range, again using ADS.

**Figure 18:**
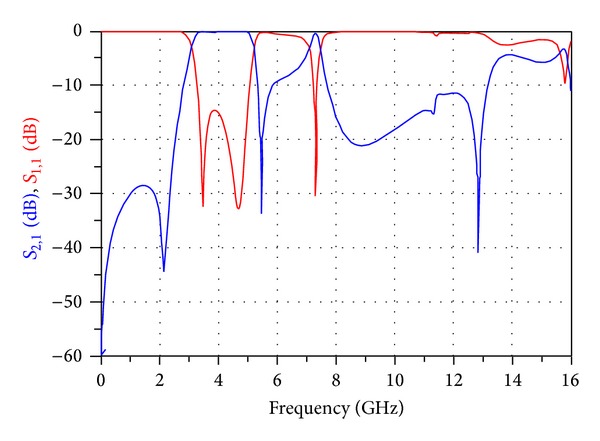
Frequency response of the simulated filter in the workspace of ADS plotted versus return losses (*S*_11_) and insertion losses (*S*_21_) in the range of 0–16 GHz.

Figure 18 shows the frequency response of the simulated filter in the workspace of ADS plotted versus return losses (*S*_11_) and insertion losses (*S*_21_) in the range of 0–16 GHz.

As can be observed in the frequency response of the filter simulated using ADS versus return losses (*S*_11_) and insertion losses (*S*_21_) in the range of 0–16 GHz shown in Figure 18, this filter has the first harmonic at about 7.5 GHz, which limits the stop-band of this filter, and therefore this filter cannot be used for usages that require greater stop-bands.

## 4. Analysis of the Structure of Newly Proposed Broadband Band-Pass Filters

**Figure 19:**
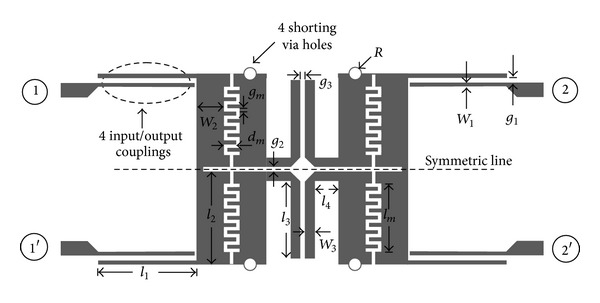
Configuration of the proposed balanced dual-band band-pass filter using coupled stepped-impedance resonators [3].

Shi and Xue (2010) [3] proposed a novel balanced dual-band band-pass filter, using coupled stepped-impedance resonators, which can greatly simplify the balanced dual-band RF front-end. The differential- and common-mode equivalent half circuits were given in their study and also a demonstration filter was implemented for WLAN application. They concluded that, by properly designing the resonators, associated with the filter composed of eight SIRs, dual-band differential-mode band-pass response can be obtained and an improved design for higher common-mode suppression level is realized by adding two open stubs; see Figure 19.

**Figure 20:**
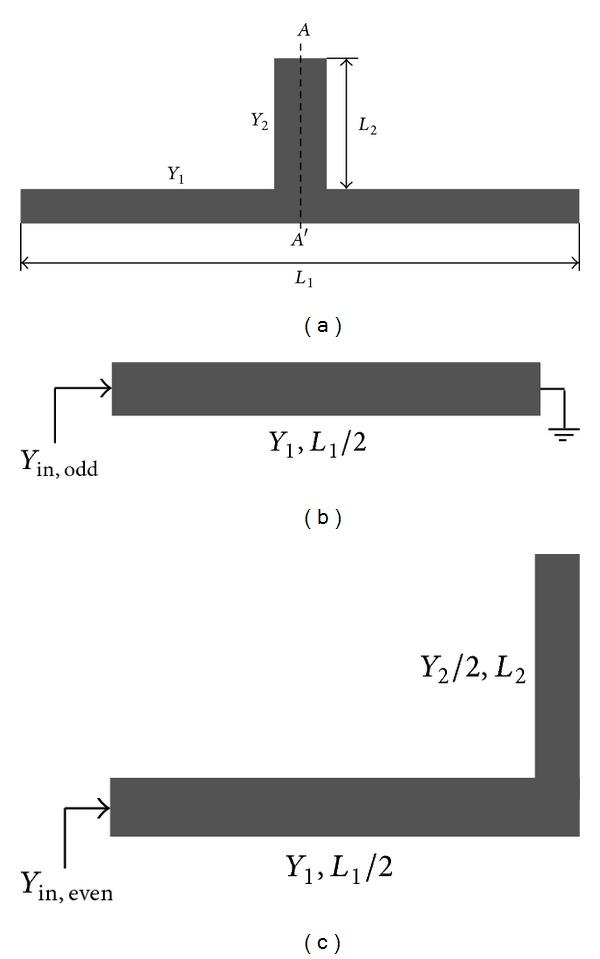
(a) Structure of the proposed stub-loaded resonator, (b) odd-mode equivalent circuit, and (c) even-mode equivalent circuit [4].

Zhang et al. [4] proposed dual-band band-pass filters using novel stub-loaded resonators which are found to have the advantage that the even-mode resonant frequencies can be flexibly controlled whereas the odd-mode resonant frequencies are fixed. Also by introducing spur-line and to improve the selectivity, they designed a filter with four transmission zeros on either side of both pass-bands based on the proposed SLR; see Figure 20.

In this paper, a new structure of these resonators will be proposed, in which, instead of the second section SIR, which had a lower impedance, a two-section SIR will be used.

**Figure 21:**
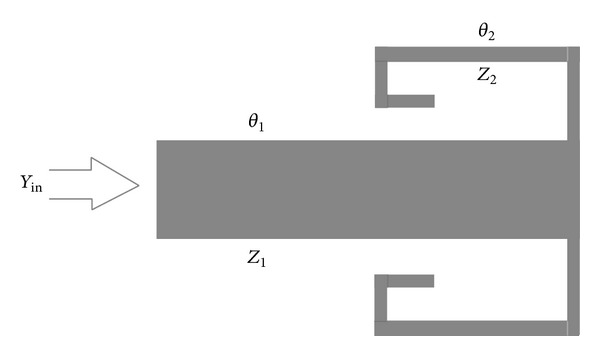
Structure and schematic diagram of the new proposed structure for SIR resonators, in which the second section of the SIR is replaced with a two branch-line section.

As can be observed in the proposed structure presented in Figure 21, the new SIR contains two sections, where the first section is identical to the old SIR and the second section has two sections with parallel stubs.

## 5. Simulation of the Structure of the Proposed Broadband Band-Pass Filter 

**Figure 22:**
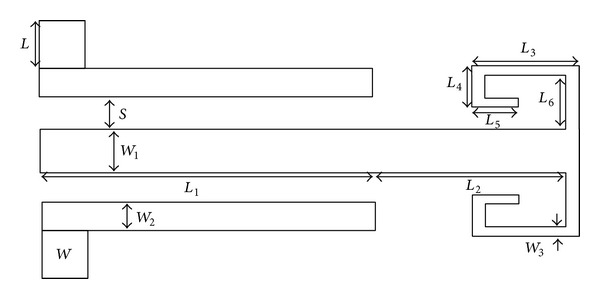
Structure of the proposed broadband band-pass filter.

As is shown in Figure 22, the structure of the band-pass filter contains a two-section SIR in which the first section is similar to the first section of regular SIRs and the second section has two equal parallel branches. Figure 22 shows the structure of a resonator which uses the combination of two transmission lines with a length of *λ*/4 as feed lines. Generally the structure combined with these feed lines will provide a wideband band-pass filter.

**Figure 23:**
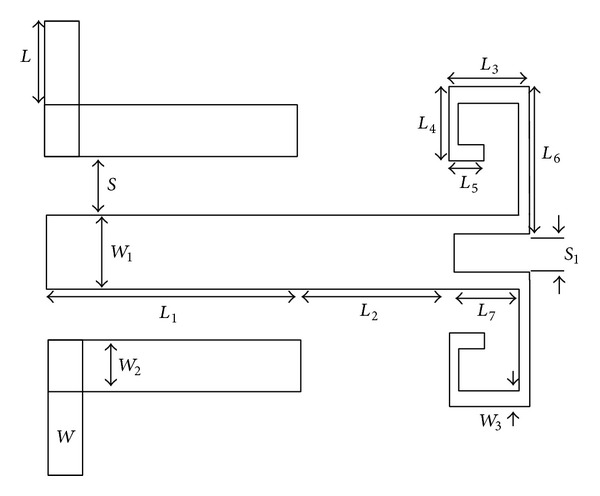
Structure of the proposed broadband band-pass filter, *L* = 2.2 mm, *L*_1_ = 12 mm, *L*_2_ = 3.4 mm, *L*_3_ = 1.6 mm, *L*_4_ = 0.5 mm, *L*_5_ = 0.4 mm, *L*_6_ = 1.4 mm, *L*_7_ = 1.2 mm,* W* = 2.2 mm, *W*_1_ = 0.3, *W*_2_ = 0.28 mm, *W*_3_ = 0.2,* S* = 0.1 mm, and *S*_1_ = 0.1 mm.

For the improvement of the above-mentioned resonator, a type of electric coupling is embedded between the cochlear branches of the SIR. The creation of this coupling in the mentioned resonator will create a notch in the ultrawide pass-band.  Figure 23 shows the structure of this with its values.

The main goal of this paper is to improve the operation of old band-pass filters by acquiring a large bandwidth and also with low insertion loss. In Figure 23 this band-pass filter based on the optimized parameters is shown using the ADS program.

**Figure 24:**
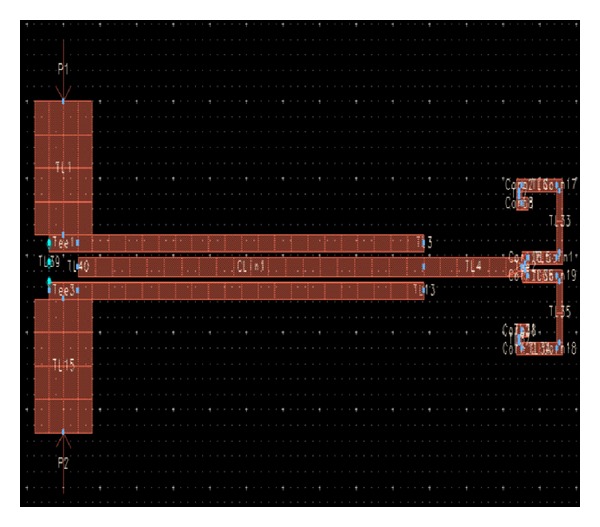
Structure of the proposed new broadband band-pass filter.

After selecting the optimized values for the filter parameters, its structure is taken to program workspace.  Figure 24 shows the structure of the designed broadband band-pass filter in the workspace of ADS.

**Figure 25:**
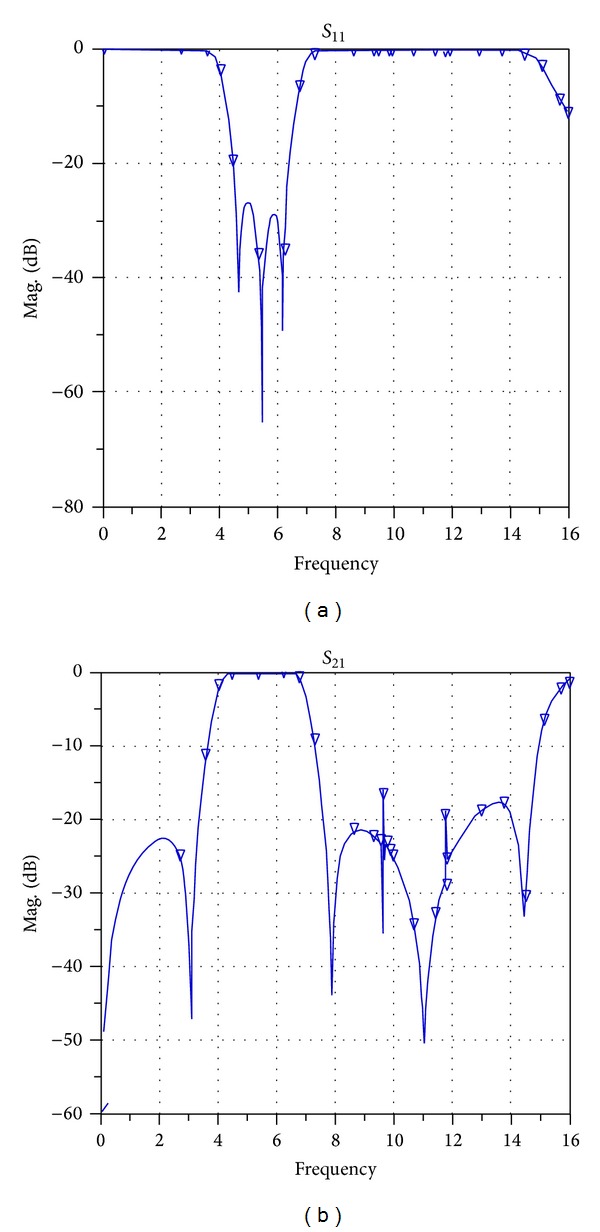
Simulated frequency response of the proposed new filter in the workspace of ADS plotted versus (a) return losses (*S*_11_) and (b) insertion losses (*S*_21_).

After implementing the filter in the workspace of ADS, the structure of this filter is implemented on a substrate with a thickness of 0.787 and a dielectric constant of 2.2 and the frequency response is simulated in the range of 0–16 GHz.  Figure 25 shows the frequency response of the simulated filter in the workspace of ADS plotted versus return losses (*S*_11_) and insertion losses (*S*_21_).

**Figure 26:**
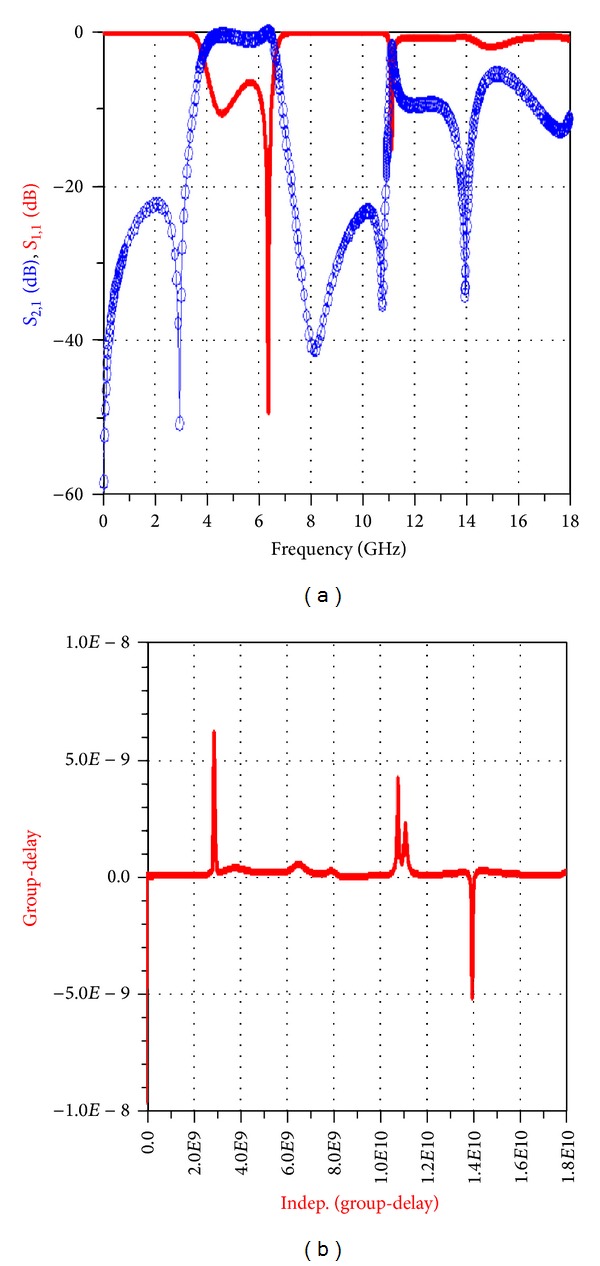
Frequency response of the simulated filter in the ADS program versus (a) return losses (*S*_11_) and insertion losses (*S*_21_) in the range of 0–16 GHz and (b) filter phase delay.

It is observed that this filter gives us a large bandwidth and also the group-delay of this band-pass filter is linear in its pass-band (see Figure 26).

## 6. Conclusion

In this paper the newest broadband band-pass designed in recent years has been studied. The two band-pass filters studied in this paper are the result of the most recent studies made in the past few years. One was designed using stepped-impedance resonators and the other was designed using multiple-mode resonators. The new model proposed in this paper is an improved model of these two resonators. In the new structure, using the zeroes of the frequency response transmission, we can improve this type of filter and, in fact, improve their selectivity.

## Figures and Tables

**Table 1:** Optimized values of the given broadband band-pass filter.

Parameter	*L*	*L*_1_	*L*_2_	*L*_3_	*L*_4_	*L*_5_
Value	1.2 mm	8.59 mm	0.3 mm	0.6 mm	5.4 mm	4.35 mm

Parameter	*L*_6_	*L*_7_	*L*_8_	*L*_9_	*W*_1_	*W*_2_

Value	4.1 mm	3.36 mm	1.7 mm	2.74 mm	0.2 mm	0.44 mm

Parameter	*W*_4_	*W*_5_	*W*_6_	*S*	*S*_1_	

Value	1.1 mm	0.1 mm	1.6 mm	0.08 mm	0.24 mm	

**Table 2:** Optimized values for the broadband band-pass filter given in Figure 8.

Parameter	*L*_1_	*L*_2_	*L*_3_	*W*_1_	*W*_2_	*S*_1_	*S*_2_
Value	9.8 mm	18.2 mm	13.4 mm	3.4 mm	0.2 mm	0.14 mm	0.4 mm
